# Persistence of pdm2009-H1N1 internal genes of swine influenza in pigs, Thailand

**DOI:** 10.1038/s41598-020-76771-2

**Published:** 2020-11-16

**Authors:** Chanakarn Nasamran, Taveesak Janetanakit, Supasama Chiyawong, Supanat Boonyapisitsopa, Napawan Bunpapong, Duangduean Prakairungnamthip, Aunyaratana Thontiravong, Alongkorn Amonsin

**Affiliations:** 1 Center of Excellence for Emerging and Re-emerging Infectious Diseases in Animals, Faculty of Veterinary Science, Chulalongkorn University, Bangkok, Thailand; 2 Department of Veterinary Public Health, Faculty of Veterinary Science, Chulalongkorn University, Bangkok, 10330 Thailand; 3 Department of Microbiology, Faculty of Veterinary Science, Chulalongkorn University, Bangkok, Thailand

**Keywords:** Microbiology, Virology

## Abstract

Swine influenza is one of the important zoonotic diseases of pigs. We conducted a longitudinal survey of swine influenza A viruses (S-IAV) circulating in a pig farm with history of endemic S-IAV infection from 2017 to 2018. The samples were collected from 436 pigs including nasal swab samples (n = 436) and blood samples (n = 436). Our result showed that 18.81% (82/436) were positive for influenza A virus and subsequently 57 S-IAV could be isolated. Then 24 out of 57 S-IAVs were selected for whole genome sequencing and could be subtyped as S-IAV-H1N1 (n = 18) and S-IAV-H3N2 (n = 6). Of 24 S-IAVs, we observed 3 genotypes of S-IAVs including rH1N1 (pdm + 1), rH1N1 (pdm + 2), and rH3N2 (pdm + 2). Since all genotypes of S-IAVs in this study contained internal genes from pdmH1N1-2009, it could be speculated that pdmH1N1-2009 was introduced in a pig farm and then multiple reassorted with endemic S-IAVs to generate diversify S-IAV genotypes. Our study supported and added the evidences that pdmH1N1-2009 and it reassortant have predominately persisted in pig population in Thailand. Thus, monitoring of S-IAVs in pigs, farm workers and veterinarians in pig farms is important and should be routinely conducted.

## Introduction

Swine influenza is one of the important zoonotic diseases of pigs. Swine influenza A virus (S-IAV) subtypes H1N1, H3N2 and H1N2 cause respiratory disease in pigs worldwide. In Thailand, S-IAV-H1N1 and S-IAV-H3N2 were first reported in the 1970s^1^. S-IAV-H1N2 was reported in pig farms in 2005^2^. In general, there are 3 major lineages of H1 gene; Classical swine lineage (CS; 1A), Eurasian avian-like swine lineage (EA; 1B) and human seasonal (Hu; 1C). The CS (1A) has 6 subclusters; α (1A.1), β (1A.2), γ1 (1A.3.3.3), γ2 (1A.3.2), TR (1A.3.3.1) and pdm09 (1A.3.3.2)^3^. Alpha subclusters (1A.1) could be further classified to subgroups; 1A1.1, 1A.1.2 and 1A.1.3. In Thailand, previous study reported that genotypes of endemic Thai-S-IAV-H1N1 contained H1 from Classical swine lineage (CS) and seven genes from Eurasian avian-like swine lineage (EA) (designated 7 + 1) or H1 and NS genes from Classical swine lineage and six genes from Eurasian avian-like swine lineage (designated 6 + 2)^4,5^. For S-IAV-H3N2, there are 2 major lineages of H3 gene; North American lineage and Eurasian swine lineage. The North American lineage can be classified into 4 clades of H3 (clade I, clade II, clade III, clade IV) as well as Human-like swine. The Clade IV of North American S-IAV can be further divided into 6 sub-clusters: A-F. In Thailand, the endemic Thai-S-IAV-H3N2 contained H3 and N2 genes from human-like swine lineage and other internal genes from Eurasian swine lineage (PB1, PB2, PA and M genes) and Classical swine lineage (NP and NS genes)^6^. For S-IAV-H1N2, endemic Thai-S-IAV-H1N2 contains N2 gene from human-like swine lineage, two segments (HA and NS) from Classical swine lineage and other five internal genes (PB2, PB1, PA, NP and M) from Eurasian swine lineage^5^.

Genetic diversity of swine influenza viruses has been observed especially in Asian countries. It has been reported that all major S-IAVs lineages from different continents (CS-H1, EA-H1, pdm2009-like H1, American TR, human-like H3, European H3N2, Avian-like viruses etc.,) are co-circulating in pigs in Asia including Thailand. Co-circulation of different virus lineages could result in virus reassortment and more genetic diverse of the viruses. After the introduction of pdmH1N1-2009, several studies reported pdmH1N1-2009 infection in pigs^7,8^. Then endemic Thai-S-IAVs were reassorted with pdmH1N1-2009, which contributing triple reassortment internal genes (TRIG). Reassortant S-IAVs with pdmH1N1-2009 became predominant genotypes of S-IAVs in pigs in Thailand and the region^9,10^. Persistence of gene especially pdmH1N1-2009 in pigs for decade could contribute the generation of a novel virus with high infectivity and more virulence in pigs or humans. Due to the novel S-IAV reassortant could be found in pig farms, routine genetic monitoring of S-IAVs is important.

It has been reported that the transmission of human-origin viruses to pigs could contribute the adaptations or mutations for the fitness of the viruses. For example, adaptive mutations of pdmH1N12009 after infection in pigs, the NP gene of the virus develop D53E mutation correlated to less resistance to the antiviral factor in pigs. While some human-origin viruses contain H289Y substitution to reduce resistance to the antiviral factor and increase viral replication^11^. Some human-origin viruses maintain or loss glycosylation sites in HA protein due to less antibody selection pressure in pigs^12^. Thus genetic analysis of the S-IAVs is important to identify the potential adaptations or mutations that might relate to potential zoonotic of the viruses. The objective of this study was to conduct a longitudinal survey of S-IAVs circulating in a pig farm with history of endemic S-IAV infection from 2017 to 2018. Genetic diversity of S-IAVs and evidence of genetic reassortment of S-IAVs in a pig farm was investigated.

## Results

### Survey for swine influenza viruses in a pig farm

In this study, we conducted longitudinal sample collection from a pig farm in central province of Thailand from 2017 to 2018. This pig farm had a history of endemic S-IAV infection in the past 5 years. Our result showed that 18.81% (82/436) and 19.95% (87/436) of nasal swab samples were positive and suspected for S-IAVs, respectively. By year, S-IAVs could be detected all year round in 2017 which highest in June 2017 (33.33%). In 2018, S-IAVs could be detected in every month of 2018, except March 2018. Highest percentage of S-IAV positivity was in April 2018 with 40.63% (13/32). The 169 S-IAV positive and suspected samples were subjected to virus isolation by egg inoculation. Our result showed that 57 influenza viruses (13.07%) could be isolated (Supplement Table 1).

### Diversity of swine influenza viruses in a pig farm

**Figure 1:**
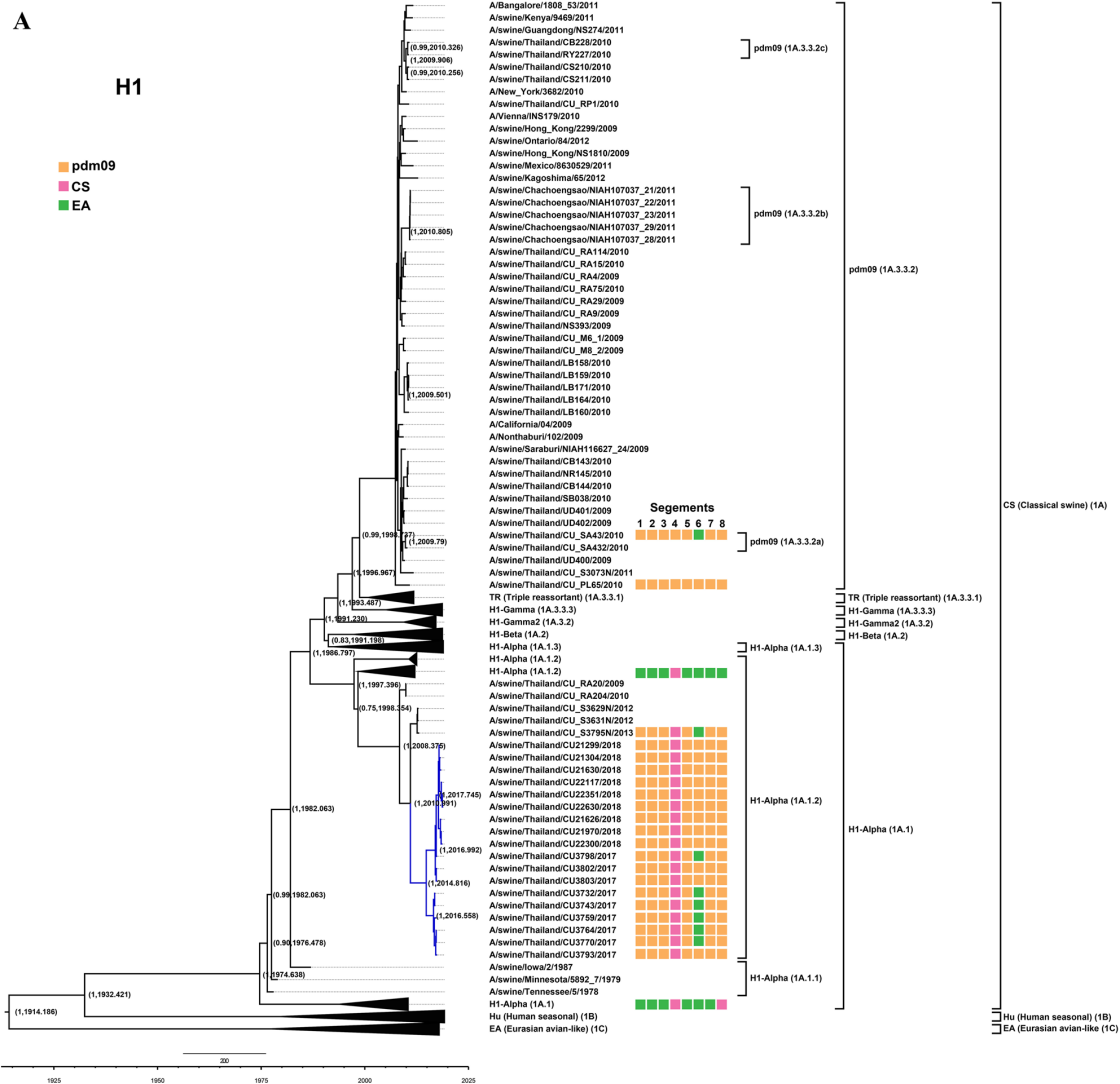

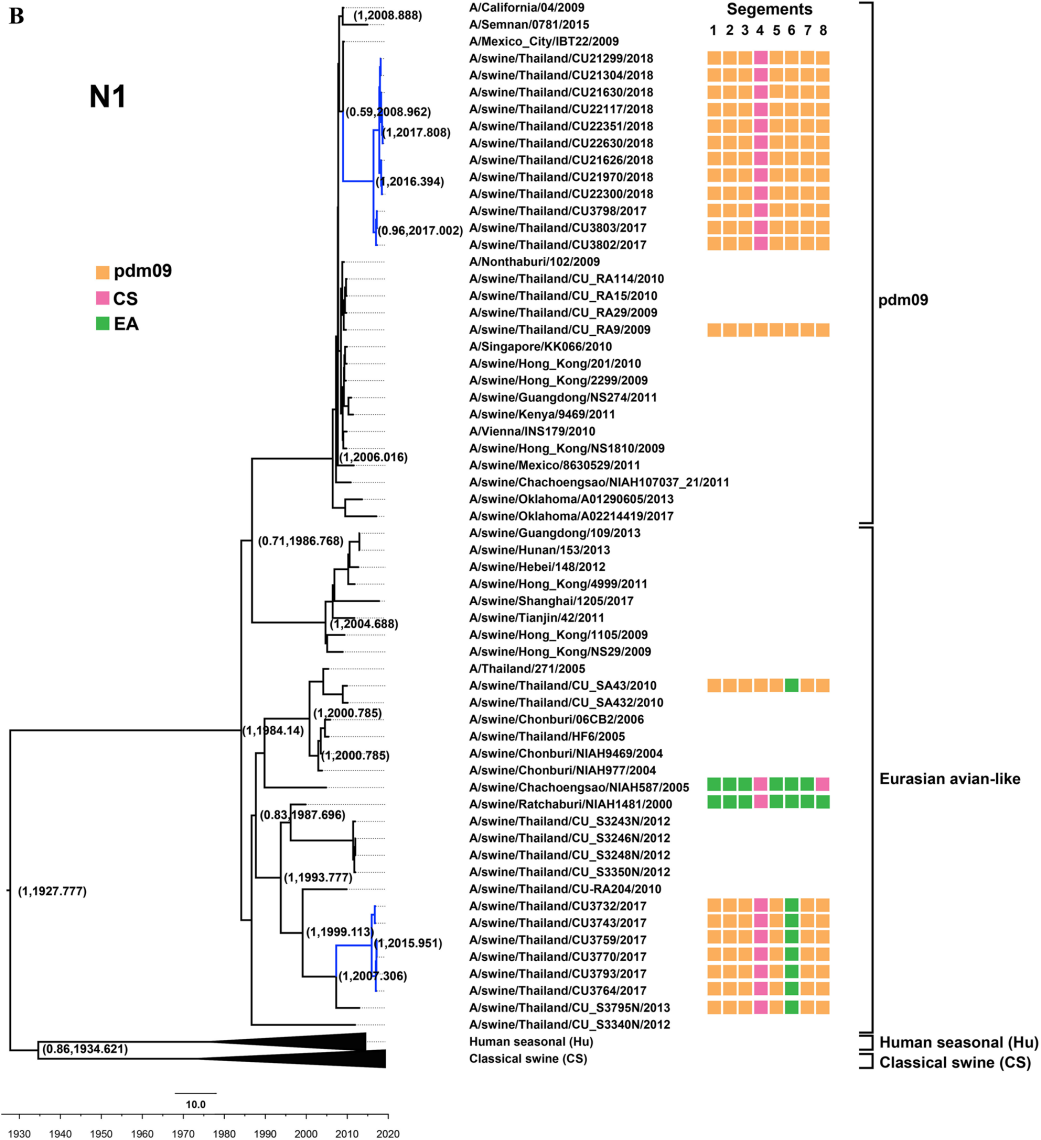
(**A**) Phylogenetic tree of H1 of S-IAV-H1N1. (**B**) Phylogenetic tree of N1 of S-IAV-H1N1. Yellow, pink, and green colors indicate pdm2009-H1N1 lineage (pdm09), classical swine lineage (CS), Eurasian avian-like lineage (EA), respectively. Circles indicate S-IAV-H1N1 isolated in this study. The phylogenetic tree was constructed by using the Beast program with Bayesian Markov‐Chain Monte Carlo (BMCMC), with 50,000,000 generations and an average standard deviation of split frequencies < 0.10. Values on branches represent posterior probability and times of most recent common ancestor (TMRCA) among H1-S-IAV.

**Figure 2:**
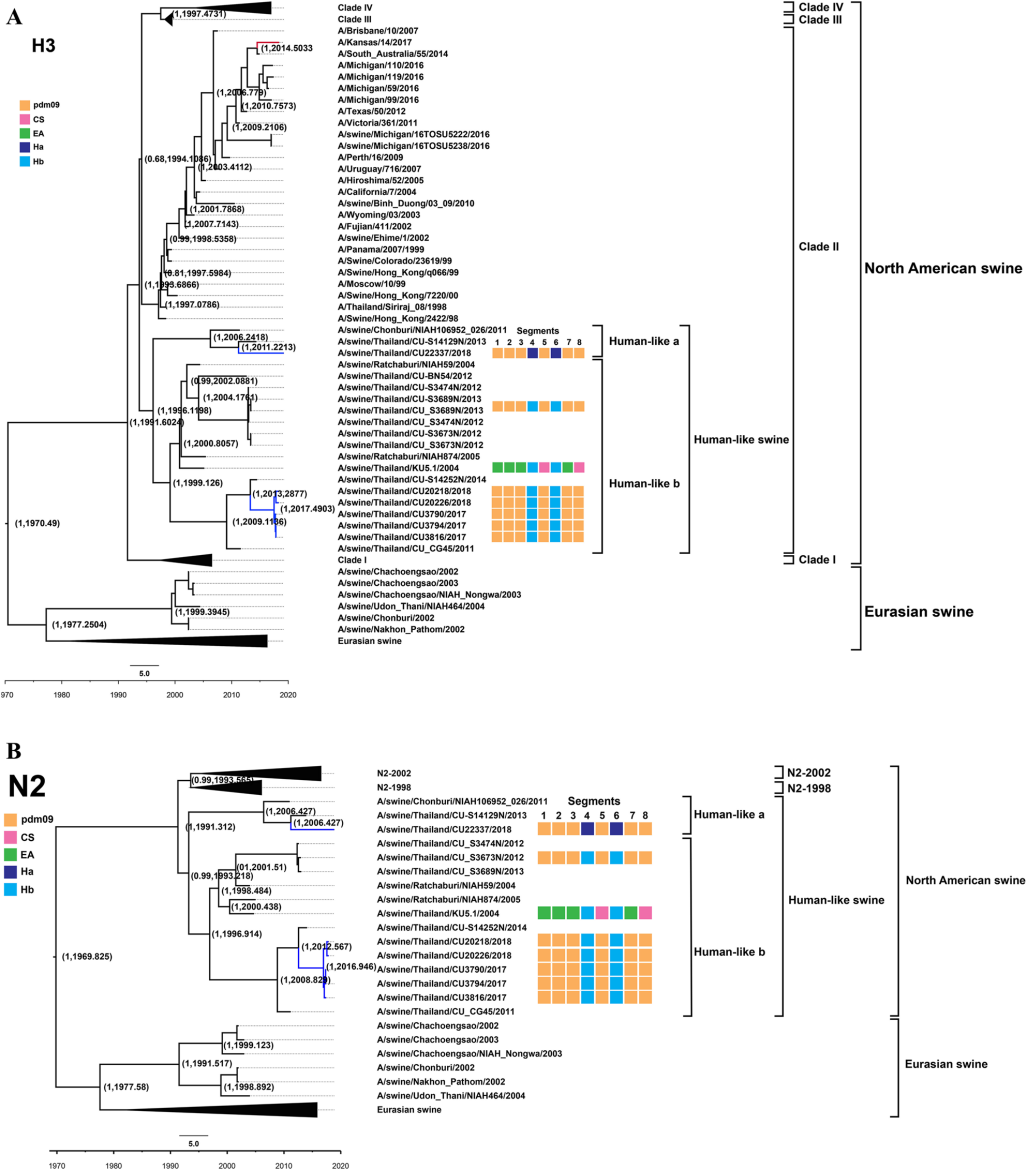
(**A**) Phylogenetic tree of H3 of S-IAV-H3N2. (**B**) Phylogenetic tree of N2 of S-IAV-H3N2. Yellow, pink, green, blue and dark blue colors indicate pdm2009-H1N1 lineage (pdm09), classical swine lineage (CS), Eurasian avian-like lineage (EA), Ha human-like swine lineage (Ha), Hb human-like swine lineage (Hb), respectively. Circles indicate S-IAV-H1N1 isolated in this study. The phylogenetic tree was constructed by using the Beast program with Bayesian Markov‐Chain Monte Carlo (BMCMC), with 50,000,000 generations and an average standard deviation of split frequencies < 0.10. Values on branches represent posterior probability and times of most recent common ancestor (TMRCA) among H3-S-IAV.

Twenty four out of 57 swine influenza viruses were selected for whole genome sequencing. The 24 S-IAVs were selected based on time of sample collection, subtypes of the viruses and virus titer (low Ct-value). To identify subtype of S-IAVs, nucleotide sequences of HA and NA genes were compared with nucleotide sequences in the NCBI database by using BLAST program. Our result showed that 24 S-IAVs could be identified as S-IAV-H1N1 (n = 18) and S-IAV-H3N2 (n = 6) (Table 1). Phylogenetic tree of H1 was constructed by comparing S-IAV-H1N1 (n = 18) to 125 references viruses. Phylogenetic tree of H1 gene showed that all S-IAV-H1N1s in this study were clustered with alpha sublineage (1A.1.2) of the classical swine lineage (Fig. 1A). It is noted that alpha sublineage (1A.1.2) is a common lineage of endemic Thai-S-IAVs. Phylogenetic tree of N1 was constructed by comparing 18 S-IAV-H1N1 to 70 references viruses. N1 of S-IAV-H1N1s in this study was clustered with either Eurasian avian-like swine lineage (n = 6) or pdm09 lineage (n = 12) (Fig. 1B). The Eurasian avian-like swine lineage is a common lineage of N1 of endemic S-IAVs in Thailand. Phylogenetic tree of H3 was constructed by comparing S-IAV-H3N2 (n = 6) to 142 references viruses. Phylogenetic analysis showed that H3 of S-IAV-H3N2 in this study was clustered with North America lineage, sublineage human-like swine (both Ha and Hb), which is previously identified as common lineages of endemic Thai-S-IAV-H3N2 (Fig. 2A). Phylogenetic tree of N2 was constructed by comparing S-IAV-H3N2 (n = 6) to 82 references viruses. The reference of NA2 gene included 2 major lineages; North American lineage and Eurasian swine lineage. The North American lineage contains 2 subclusters: N2-1998 and N2-2002 lineages from human seasonal influenza. Phylogenetic analysis showed that N2 genes of S-IAV-H3N2s were clustered into North American lineage with sublineage human-like swine of endemic Thai S-IAV-H3N2 (Fig. 2B). Phylogenetic trees of the internal genes of the S-IAV-H1N1 and S-IAV-H3N2 were constructed by comparing S-IAV-H1N1 (n = 18) and S-IAV-H3N2 (n = 6) to references viruses. The reference viruses included Classical swine lineage, Eurasian swine lineage, North America triple reassortant lineage, human seasonal lineage and pdm09 lineage. Phylogenetic analysis of each internal genes showed that internal genes of S-IAV-H1N1s (n = 18) and S-IAV-H3N2s (n = 6) were clustered with pdm09 lineage indicating S-IAVs in this study acquired internal genes (backbone) from pdmH1N1-2009 virus (Supplement Figures 1–6).

**Table 1:** Description of Thai S-IAV-H1N1 and S-IAV-H3N2 characterized in this study.

Virus	Subtype^a^	Year	Host	Age	GenBank no
**S-IAV-H1N1**					
A/swine/THA/CU3732/2017	rH1N1 (pdm + 2)	Jan-17	Swine	4 weeks	MT378014-21
A/swine/THA/CU3743/2017	rH1N1 (pdm + 2)	Jan-17	Swine	6 weeks	MT378022-29
A/swine/THA/CU3759/2017	rH1N1 (pdm + 2)	Apr-17	Swine	4 weeks	MT378030-37
A/swine/THA/CU3764/2017	rH1N1 (pdm + 2)	Apr-17	Swine	6 weeks	MT378038-45
A/swine/THA/CU3770/2017	rH1N1 (pdm + 2)	Apr-17	Swine	6 weeks	MT378046-53
A/swine/THA/CU3793/2017	rH1N1 (pdm + 2)	Jun-17	Swine	4 weeks	MT378062-69
A/swine/THA/CU3802/2017	rH1N1 (pdm + 1)	Jun-17	Swine	6 weeks	MT378086-93
A/swine/THA/CU3803/2017	rH1N1 (pdm + 1)	Jun-17	Swine	6 weeks	MT378094-01
A/swine/THA/CU3798/2017	rH1N1 (pdm + 1)	Jun-17	Swine	4 weeks	MT378078-85
A/swine/THA/CU21299/2018	rH1N1 (pdm + 1)	Apr-18	Swine	6 weeks	MT377918-25
A/swine/THA/CU21304/2018	rH1N1 (pdm + 1)	Apr-18	Swine	10 weeks	MT377942-49
A/swine/THA/CU21626/2018	rH1N1 (pdm + 1)	Jun-18	Swine	6 weeks	MT377950-57
A/swine/THA/CU21630/2018	rH1N1 (pdm + 1)	Jun18	Swine	8 weeks	MT377958-65
A/swine/THA/CU21970/2018	rH1N1 (pdm + 1)	Jul-18	Swine	6 weeks	MT377966-73
A/swine/THA/CU22117/2018	rH1N1 (pdm + 1)	Aug-18	Swine	4 weeks	MT377974-81
A/swine/THA/CU22300/2018	rH1N1 (pdm + 1)	Sep-18	Swine	10 weeks	MT377982-89
A/swine/THA/CU22351/2018	rH1N1 (pdm + 1)	Oct-18	Swine	9 weeks	MT377998-05
A/swine/THA/CU22630/2018	rH1N1 (pdm + 1)	Nov-18	Swine	4 weeks	MT378006-13
**S-IAV-H3N2**					
A/swine/THA/CU3794/2017	rH3N2 (pdm + 2)	Jun-17	Swine	4 weeks	MT378102-09
A/swine/THA/CU3816/2017	rH3N2 (pdm + 2)	Jun-17	Swine	10 weeks	MT378070-77
A/swine/THA/CU3790/2017	rH3N2 (pdm + 2)	Jun-17	Swine	4 weeks	MT378054-61
A/swine/THA/CU20218/2018	rH3N2 (pdm + 2)	Nov-17	Swine	8 weeks	MT377926-33
A/swine/THA/CU20226/2018	rH3N2 (pdm + 2)	Nov-17	Swine	12 weeks	MT377934-41
A/swine/THA/CU22337/2018	rH3N2 (pdm + 2)	Oct-18	Swine	6 weeks	MT377990-97

^a^rH1N1 (pdm + 1): reassorted S-IAV-H1N1 genotype contained HA gene from classical swine lineage (CS) and other genes from pandemicH1N1-2009 lineage (pdm09). rH1N1 (pdm + 2): reassorted S-IAV-H1N1 genotype contained HA gene from classical swine lineage (CS), NA gene from Eurasian avian-like lineage (EA) and internal genes from pandemicH1N1-2009 lineage (pdm09). rH3N2 (pdm + 2): reassorted S-IAV-H3N2 genotype contained HA and NA genes from human-like swine and internal genes from pandemicH1N1-2009 lineage (pdm09).

**Figure 3:**
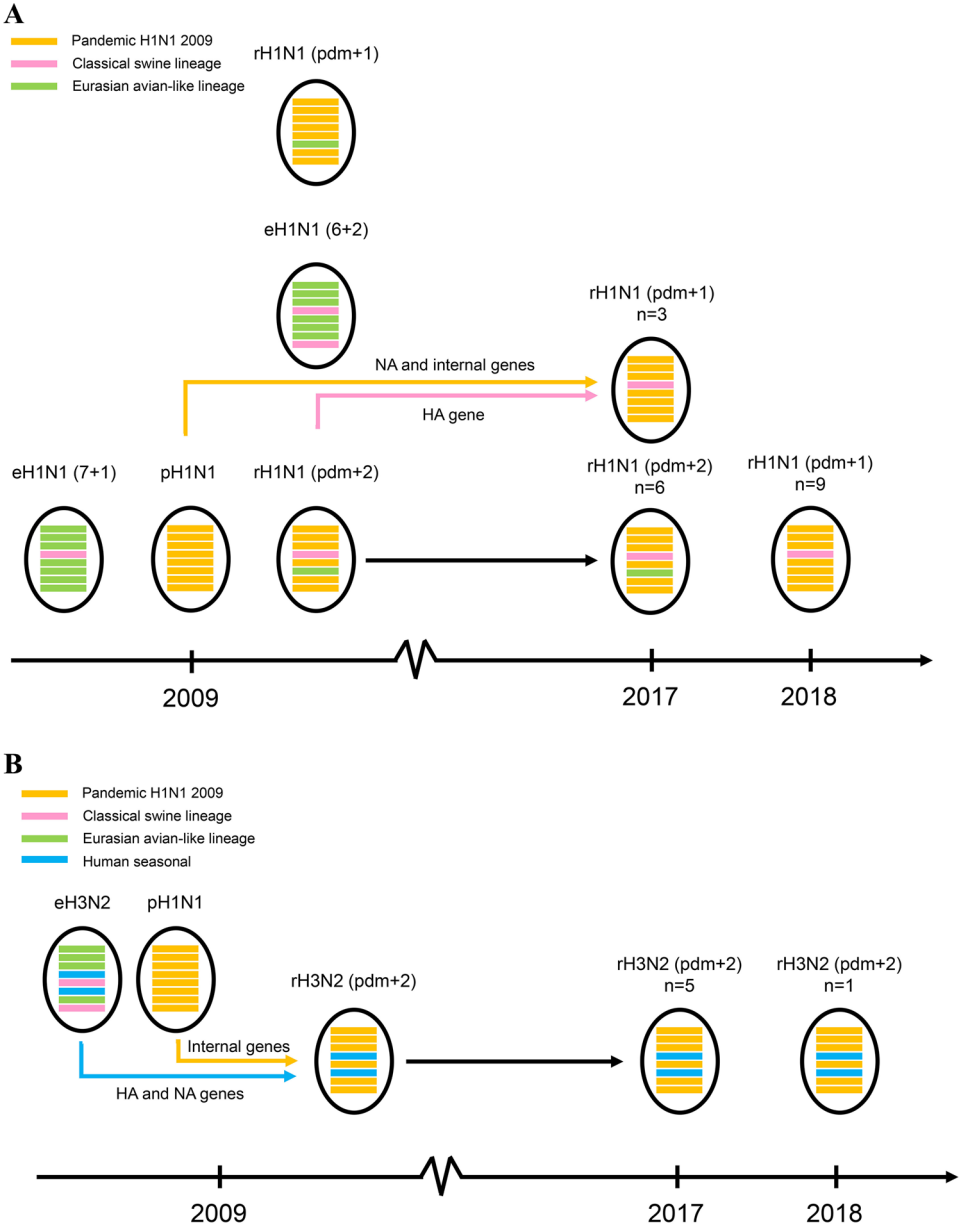
(**A**) Schematic presentation of genotypes of S-IAV-H1N1 in this study. (**B**) Schematic presentation of genotypes of S-IAV-H3N2 in this study. Oval represent the virus with 8 gene segments. Lines from top to bottom represent PB2, PB1, PA, HA, NP, NA, M and NS genes. Yellow, pink, green, blue and dark blue colors indicate pdm2009-H1N1 lineage (pdm09), classical swine lineage (CS), Eurasian avian-like lineage (EA), Ha human-like swine lineage (Ha), Hb human-like swine lineage (Hb), respectively.

Gene constellation of the S-IAVs was identified by designated by using the combination of eight lineages or clusters of the virus. Of 24 S-IAVs, we observed 3 genotypes of S-IAVs including (1) rH1N1 (pdm + 1) contained H1 from classical swine lineage (CS) of endemic Thai-S-IAVs and the other 7 genes (NA and internal genes) from pdm09 lineage. (2) rH1N1 (pdm + 2) contained H1 from classical swine lineage (CS) of endemic Thai-S-IAVs, while N1 from Eurasian avian-like swine lineage (EA) of endemic Thai-S-IAVs and the 6 internal genes from pdm09 lineage, and (3) rH3N2 contained HA3 and NA2 genes from human-like swine lineage of endemic Thai-S-IAVs and internal genes from pdm09 lineage (Fig. 3A,B). Our result suggested that internal genes of pdm09 lineage persist and become predominant lineage in endemic Thai-S-IAVs (Figs. 1A and 2A, Supplement Table 2). It should be concerned that persistence of gene especially pdmH1N1-2009 in pigs for long period in the pig farms could contribute the generation of a novel virus with high infectivity and transmissibility in pigs or humans.

For genetic analysis of H1 cleavage site, S-IAVs in this study had identical H1 cleavage site “PSIQSRGLF” to pdmH1N1-2009 and endemic S-IAVs. For receptor binding sites (HA-190, 225), most S-IAVs contained 190D and 225D suggesting preferential binding to human receptor (2,6 sialic acid receptor)^13^. Expect one virus (CU21626) contained E190 suggesting the virus prefer to bind avian receptor (2,3 sialic acid receptor)^14^. Genetic analysis of H1 antigenic sites (Sa, Sb, Ca1, Ca2 and Cb) showed that S-IAVs in this study contain similar amino acids of antigenic sites to endemic Thai-S-IAVs (Table 2). For HA3 genetic analysis, the receptor binding sites (HA3-226 and 228) of S-IAVs in this study and endemic Thai-S-IAVs posed I226 and S228 suggesting preferential binding to human receptor^14,15^. Genetic analysis of antigenic sites of H3 (A, B, C, D, and E) showed that antigenic sites A (140–146) and B (156–161, 189–199) contained amino acids similar to endemic Thai-S-IAVs (Table 3). For genetic analysis of some internal genes (PB2, M and NS genes), all S-IAVs in this study possessed glutamic acid (E) at position 627 in PB2 (E627), which correlated with less virulence of the viruses. While all S-IAVs contained aspartic acid (D) at position 701 in PB2 (N701D) and position 92 in NS (E92D), which correlated to the more virulence of the viruses^15–17^. For genetic analysis of antiviral resistance in M gene at position 31 of M gene, all S-IAVs in this study possessed amino acid N at position 31 (31N) suggesting the viral resistance to amantadine^18^. On the other hand, genetic analysis of antiviral resistance (oseltamivir) in NA gene (NA1-119, 275, 293, and 295 and NA2-146, 219, and 272) showed that all S-IAVs in this study are sensitive to oseltamivir (for NA1; E119, H275, R293, N295 and NA2; N146, S219, A272) (data not shown).

**Table 2:** Genetic analysis of the H1 gene of Thai S-IAV-H1N1 in this study.

Viruses	Subtype	H1 Cluster	H1 gene^a^												
			Antigenic site										Receptor binding site		HA cleavage site
			Sa			Sb	Ca1			Ca2		Cb	190	225	325–333
			128–129	156–160	162–167	187–198	169–173	206–208	238–240	140–145	224–225	78–83			
**Reference**															
California/04/2009	pdmH1N1	pdm	PN	KKGNS	PKLSKS	TSADQQSLYQNA	INDKG	GSS	EPG	PHAGAK	RD	SLSTAS	D	D	PSIQSRGLF
THA/CUPL65/2010	PdmH1N1	pdm	PN	KKGNS	PKLSKS	TSADQQSLYQNA	INDKG	GSS	EPG	PHAGAE	RD	SLSTAS	D	D	PSIQSRGLF
THA/CU3340N/2012	eH1N1 (6 + 2)	CS	PN	KKGNS	PKLSKS	TSTDQQSLYQNA	VNNKK	SSS	EPG	HYAGAN	RD	LLFKAS	D	D	PSIQSRGLF
THA/NIAH587/2005	eH1N1 (7 + 1)	CS	PN	KKGNS	PKLSKS	TNTDQQSLYQNA	VNNKK	GSS	EPG	PYAGAN	RG	LLFAIN	D	G	PSIQSRGLF
THA/CU3795N/2013	rH1N1 (6 + 2)	CS	PN	KKENS	PKISKS	TSNDQQSLYQNA	FNNKG	SSS	KPG	PYAGAN	RD	LLFNAS	D	D	PSIQSRGLF
THA/CUSA43/2010	rH1N1 (7 + 1)	pdm	PN	KKGNS	PKLSKS	TSADQQSLYQNA	INDKG	GSS	EPG	PHAGAK	RD	SLSTAS	D	D	PSIQSRGLF
THA/CUS3629N/2012	rH1N1 (pdm + 2)	CS	PN	KKENS	PKISKS	TSNDQQSLYQNA	FNNKG	SSS	KPG	PYAGAN	RD	LLFNAS	D	D	PSIQSRGLF
**This study**															
THA/CU3759/2017	rH1N1 (pdm + 2)	CS	PN	KKENS	PKLSKS	TSNDQQVLYQNA	FNNKG	SSS	KPE	PYAGAN	*RN*	SLFNAS	D	*N*	PSIQSRGLF
THA/CU3764/2017	rH1N1 (pdm + 2)	CS	PN	KKENS	PKLSKS	TSNDQQVLYQNA	FNNKG	SSS	KPE	PYAGAN	RD	SLFNAS	D	D	PSIQSRGLF
THA/CU3770/2017	rH1N1 (pdm + 2)	CS	PN	KKENS	PKLSKS	TSNDQQVLYQNA	FNNKG	SSS	KPE	PYAGAN	RD	SLFNAS	D	D	PSIQSRGLF
THA/CU3793/2017	rH1N1 (pdm + 2)	CS	PN	KKENS	PKLSKS	TSNDQQVLYQNA	FNNKG	SSS	KPE	PYAGAK	RD	SLFNAS	D	D	PSIQSRGLF
THA/CU3802/2017	rH1N1 (pdm + 1)	CS	PN	KKENS	PKLSKS	TSNDQQVLYQNA	FNNRG	SSS	KPE	PYAGAN	RD	LLFNAS	D	D	PSIQSRGLF
THA/CU3798/2017	rH1N1 (pdm + 1)	CS	PN	KKENS	PKLSKS	TSNDQQVLYQNA	FNNRG	SSS	KPE	PYAGAN	RD	LLFNAS	D	D	PSIQSRGLF
THA/CU3803/2017	rH1N1 (pdm + 1)	CS	PN	KKENS	PKLSKS	TSNDQQVLYQNA	FNNRG	SSS	KPE	PYAGAN	RD	LLFNAS	D	D	PSIQSRGLF
THA/CU21299/2018	rH1N1 (pdm + 1)	CS	PN	KKENS	PKLSKS	TSNDQQVLYQNA	FNNRG	SSS	KPE	PYAGAK	RD	LLFNAS	D	D	PSIQSRGLF
THA/CU21304/2018	rH1N1 (pdm + 1)	CS	PN	KKENS	PKLSKS	TSNDQQVLYQNA	FNNRG	SSS	KPE	PYAGAN	RD	LLFNAS	D	D	PSIQSRGLF
THA/CU21626/2018	rH1N1 (pdm + 1)	CS	PN	KKENS	PKLSKS	TSNEQQVLYQNA	FNNRG	SSS	KPE	PYAGAN	RD	LLFNAS	*E*	D	PSIQSRGLF
THA/CU21970/2018	rH1N1 (pdm + 1)	CS	PN	KKENS	PKLSKS	TSNDQQVLYQNA	FNNRG	SSS	KPE	PYAGAN	RD	LLFNAS	D	D	PSIQSRGLF
THA/CU22117/2018	rH1N1 (pdm + 1)	CS	PN	KKENS	PKLSKS	TSNDQQVLYQNA	FNNRG	SSS	KPE	PYAGAN	RD	LLFNAS	D	D	PSIQSRGLF
THA/CU21630/2018	rH1N1 (pdm + 1)	CS	PN	KKENS	PKLSKS	TSNDQQVLYQNA	FNNRG	SSS	KPE	PYAGAN	RG	LLFNAS	D	G	PSIQSRGLF
THA/CU22300/2018	rH1N1 (pdm + 1)	CS	PN	KKENS	PKLSKS	TSNDQQVLYQNA	FNNRG	SSS	KPE	PYAGAN	*RN*	LLFNAS	D	*N*	PSIQSRGLF
THA/CU22351/2018	rH1N1 (pdm + 1)	CS	PN	KKENS	PKLSKS	TSNDQQVLYQNA	FNNRG	SSS	KPE	PYAGAN	RD	LLFNAS	D	D	PSIQSRGLF
THA/CU22630/2018	rH1N1 (pdm + 1)	CS	PN	KKENS	PKLSKS	TSNDQQVLYQNA	FNNRG	SSS	KPE	PYAGAN	RG	LLFNAS	D	G	PSIQSRGLF
THA/CU3732/2017	rH1N1 (pdm + 1)	CS	PN	KKENS	PKLSKS	TSNDQQVLYQNA	FNNKG	SSS	KPE	PYAGAN	RG	SLFNAS	D	*G*	PSIQSRGLF
THA/CU3743/2017	rH1N1 (pdm + 1)	CS	PN	KKENS	PKLSKS	TSNDQQVLYQNA	FNNKG	SSS	KPE	PYAGAN	RD	SLFNAS	D	D	PSIQSRGLF

^a^Amino acid positions are based on H3 numbering.

**Table 3:** Genetic analysis of the H3 gene of Thai S-IAV-H3N2 in this study.

Viruses	Subtype	H3 Cluster	H3 gene^a^								
			Antigenic site							Receptor binding site	
			A	B		C	D	E		226	228
			140–146	156–161	189–199	277–282	205–221	171–175	243–249		
**Reference**											
THA/KU5.1/2004	eH3N2	Hu	KRGSVKS	KLDYKY	SDQTNLYVQAS	CNSECI	STKRSQQTVIPNIGSRP	NDKFD	LLINSTG	I	S
THA/CUS3673N/2012	rH3N2 (pdm + 2)	Hu	KRGSVKS	KLDYKY	NDQTNLYVQAS	CNYGCI	STKRSQQTVIPNIGSRP	NDKFN	LLINSTG	I	S
THA/CUS14252N/2014	rH3N2 (pdm + 2)	Hu	KRGSVKS	KLDYKY	SDQTNLYVQAS	CNSECI	STKRSQQTVIPNIGFRP	NDKFD	LLINSTG	I	S
THA/CUS14129N/2013	rH3N2 (pdm + 2)	Hu	KRGYVNS	QSGHKY	SDQTSLYVQAS	CNSECV	STKRSQQTVIPNIGSRP	NEKFD	LLINSTG	I	S
**This study**		Hu									
THA/CU3794/2017	rH3N2 (pdm + 2)	Hu	KRGSVKS	QLNYKY	SDQTNLYVQAS	CNSECI	STKRSQQTVIPNIGFRP	NDKFD	LLINSTG	I	S
THA/CU3816/2017	rH3N2 (pdm + 2)	Hu	KRGSVKS	QLNYKY	SDQTNLYVQAS	CNSECI	STKRSQQTVIPNIGFRP	NDKFD	LLINSTG	I	S
THA/CU3790/2017	rH3N2 (pdm + 2)	Hu	KRGSVKS	QLNYKY	SDQTNLYVQAS	CNSECI	STKRSQQTVIPNIGFRP	NDKFD	LLINSTG	I	S
THA/CU20226/2017	rH3N2 (pdm + 2)	Hu	KRGSVKS	QLNYKY	SDQTNLYVQAS	CNSECI	STKRSQQTVIPNIGFRP	NDKFD	LLINSTG	I	S
THA/CU20218/2017	rH3N2 (pdm + 2)	Hu	KRGSVKS	QLNYKY	SDQTNLYVQAS	CKSECI	STKRSQQTVIPNIGFRP	NDKFD	LLINSKG	I	S

^a^Amino acid positions are based on H3 numbering.

For serological test of S-IAVs, the serum samples were tested for antibodies against influenza subtypes H3N2, H1N1 and pdmH1N1. Our results showed that serum samples had antibodies against S-IAV-H3N2 (37.23%; 140/436), S-IAV-H1N1 (18.35%; 69/436) and pdmH1N1 viruses (27.13%; 102/436). In detail, the HI titer against S-IAV-H3N2 (endemic Thai-S-IAVs-H3N2) was highest in April 2017 (73.33%) and in September 2018 (51.52%). For S-IAV-H1N1, antibody titer against endemic Thai-S-IAV-H1N1 was highest in November 2017 (16.67%) and in August 2018 (29.03%). For pdmH1N1, HI titer against pH1N1 was highest in November 2017 (50.00%) and in July 2018 (63.33%).

## Discussion

In this study, we conducted a longitudinal survey in a pig farm from January 2017 to November 2018. The previous study on S-IAVs in this pig farm in 2015 showed that this pig farm had been infected with S-IAVs and estimated prevalence of S-IAVs was 6.66%^5^. Comparing to previous study, the S-IAV prevalence in this study was 18.81% (from January 2017 to November 2018), which was higher than previous report in 2015. The possible explanation is that this study was more focusing on target sample collection in piglets and weaning pigs (4–10 week-old), thus higher prevalence of S-IAVs was observed.

In this study, two subtypes of S-IAVs (S-IAV-H1N1 and S-IAV-H3N2) were identified. Comparing to the previous study in Thailand during 2000–2014, three subtypes of S-IAVs were identified, but S-IAV-H1N2 could not be identified in this study. Notably, S-IAV-H1N2 has lower prevalence than S-IAV-H1N1 and S-IAV-H3N2^5,19^. Phylogenetic analysis of H1 gene showed that S-IAVs in this study as well as endemic Thai-S-IAVs belonged to the distinct sublineage 1A.1.2 of classical swine (CS)^5,9^. Based on the MCC phylogenetic tree, the H1 gene of S-IAVs in this study was estimated to have separated from endemic S-IAVs-H1N1 since 2010 (Fig. 1A). While N1 gene of S-IAVs in this study was closely related to either Eurasian avian-like swine lineage (EA) of endemic Thai-S-IAVs or pdm09 lineage of pdmH1N1-2009 virus. This result suggested that pdmH1N1-2009 circulated for a period of time or repeat introduced into this pig farm. Then the pdmH1N1-2009 reassorted with other endemic S-IAVs and contribute N1 gene to novel reassortant influenza viruses. Phylogenetic analysis of H3 and N2 genes showed that H3 and N2 were clustered with human-like swine viruses that introduced in pigs during 1990s and became the predominant subclusters (Ha and Hb) of endemic S-IAV-H3N2 in Thailand^20^. In this study, HA3 and NA2 genes were still clustered with human-like swine lineage, subclusters Ha (n = 1) and Hb (n = 5) suggesting the fitness combination of surface genes (H3 and N2) from endemic S-IAVs and internal genes from pdmH1N1-2009^21^. The MCC tree of H3 showed that the S-IAVs in this study was diverged from the endemic S-IAVs-H3N2 since 2012–2013.

In this study, there were 3 reassortant genotypes of S-IAVs; rH1N1 (pdm + 1), rH1N1 (pdm + 2) and rH3N2 (pdm + 2). In Thailand, at least 7 genotypes have been reported including eH1N1 (6 + 2), eH1N1 (7 + 1), rH1N1 (pdm + 1), rH1N1 (pdm + 2), rH1N2 (pdm + 2), rH3N2 (pdm + 2) and pdmH1N1 (Supplement Table 2)^5,9,20^. The genotype rH1N1 (pdm + 2) and rH3N2 (pdm + 2), which identified in this study, have been previously reported in Thailand indicating these genotypes are stable and continuous circulating in a pig farm for several years. In contrast, novel genotype rH1N1 (pdm + 1), (H1 from classical swine and other 7 genes from pdmH1N1), has never been reported in Thailand. Our result supported the reassortment or multiple-reassortment between endemic Thai-S-IAVs and pdmH1N1-2009. Reassortment of swine influenza viruses have been observed worldwide such as in China^22,23^, Europe^24^ and America^25–27^. Since all genotypes of S-IAVs in this study contain backbone (PB2, PB1, PA, NP, M and NS genes) from pdmH1N1-2009. It could be speculated that the entire pdmH1N1-2009 virus did not persist but the internal genes of pdmH1N1-2009 have became predominant lineage of S-IAVs in Thailand. Thus the predominant of internal genes of pdmH1N1-2009 could stimulate the expand diversity and rapid evolution of S-IAVs in Thailand as well as in the region^21^.

The recent study in China has reported that the predominant reassortant S-IAVs genotype G4 (H1N1 gene from Eurasian Avian-like (HA, NA) and internal genes from pdmH1N1-2009 (PB2, PB1, PA, NP, M) and Triple reassortant internal gene (NS)) showed efficient infectivity and high pathogenicity in experimental animal model. Their findings raise a concern of potential pandemic of the viruses^28^.

Based on genetic analysis, the transmission of human-origin viruses to pigs could contribute the adaptations or mutations for the fitness of the viruses. In this study, HA cleavage site, receptor binding sites and antigenic sites of S-IAVs in this study resemble to human viruses, thus the S-IAVs could possibly infect and/or replicate in mammal host including humans. For example, outbreaks of swine origin-H3N2v from pigs to humans and humans to pigs have been documented^29^. In this study, antibodies against H1N1, H3N2 and pdmH1N1 were observed in this pig farm. It is noted that pig had antibody against pdmH1N1, even though the pdmH1N1 viruses could not be identified in the pig farm. It has been reported that no cross-reaction between antibodies against H1-S-IAV and H1 pandemic viruses^30^. Thus, the HI titer result confirmed that pigs in this farm were exposed to both endemic S-IAV-H1 and S-IAV-H3 and pdmH1N1. It should be noted that the HI antibody titer against pdmH1N1 could be either from pandemic HA already circulating in this swine farm or from pandemic HA that has been recently introduced from human. Based on our findings, we can speculate that reverse zoonotic infection from workers to pigs as well as zoonotic infection from pigs to workers in the pig farm could be occurred. Thus, monitoring of influenza virus in pigs and workers is important and should be routinely conducted. The management in pig farm is important for example personal hygiene, personal protective equipment (PPE), seasonal influenza vaccination should be practiced and used in the pig farms for prevention and control of influenza transmission in pigs and humans.

## Materials and methods

### A longitudinal survey of swine influenza viruses in a pig farm

In this study, a longitudinal sample collection was conducted in a pig farm from 2017 to 2018. A pig farm was selected based on a history of S-IAV subtypes H1N1, H1N2 and H3N2 circulated in a pig farm^5^. The pig farm is located in central province, where considered as high-density pig production area in Thailand. The pig farm is a large-scale pig farm contains approximately 1600 sow and produces 2000 piglets per month with 43 building as farm office, 32 pig housing and 10 worker housing. The farm has open-housing system with moderate biosecurity, which birds and domestic animals can access to pig housing areas. The samples were collected from 436 pigs, including nasal swab samples (n = 436) and blood samples (n = 436) from piglets and nursery pigs (4–10-week-old) with clinical signs of S-IAV infection such as coughing, sneezing and nasal discharge. Samples collection was carried out every 4 months in 2017 and every month in 2018 (Supplement Table 1). The nasal swabs were placed in viral transport media (MEM with 7% BSA, 100 U/ml penicillin, 100 mg/ml streptomycin and 1 mg/ml trypsin). The blood samples were withdraw from jugular vein and placed in 5 ml tubes. The samples were kept on ice and transported to the laboratory within 24 h. The Chulalongkorn University, Animal Care and Uses Protocol committee approved the animal study (CU-VET IACUC #1831103). All animal study procedures were performed in accordance with CU-VET IACUC guidelines and regulations.

### Detection and isolation of swine influenza virus

Viral RNA was extracted from nasal swab samples (n = 436) by using GeneAll GENTi Viral DNA/RNA Extraction Kit (GeneAll; Lisbon, Portugal) on a Genti (GeneAll; Lisbon, Portugal). For influenza A virus detection, one-step Real-time RT-PCR was performed by using SuperScript III Platinum One-Step Quantitative RT-PCR System (Invitrogen; California, USA). The one-step Real-time RT-PCR protocol with M gene specific primers and probes was carried out as previously descripted^31^. Real-time RT-PCR result was interpreted by cycle threshold (Ct- value) of < 36 as positive, Ct-value of 36–40 as suspected, and Ct-value of > 40 as negative.

For S-IAV isolation, the real-time RT-PCR positive (n = 82) and suspected (n = 51) samples were subjected to virus isolation by egg inoculation following WHO recommendation^32^. For egg inoculation, each 100 ul of nasal swab sample was inoculated into 3 embryonated chicken eggs (9 to 11-day-old). After 72 h incubation at 37 °C, allantoic fluid was collected and tested for hemagglutinating activity by HA test. For HA test interpretation, sample with HA titer of ≥ 4 HA unit per 50 µl was interpret positive. For virus confirmation, RNA samples were tested for influenza virus by using one-step Real-time RT-PCR.

### Characterization of swine influenza virus

For genetic characterization of S-IAVs, 24 IAVs were subjected to whole genome sequencing by next-generation sequencing (NGS). The criteria of selection of 24 S-IAVs for characterization were based on time of sample collection, subtypes of S-IAVs and high virus titer (low Ct-value). To perform whole genome sequencing, eight segments of S-IAVs were amplified by using one-step RT-PCR with SuperScript III RT-PCR system with Plantinum Taq DNA polymerase (Invitrogen; California, USA) with MBT12 and MBT13 primers^33^. Purified PCR products were quantified and submitted to Novogene co. LTD for Illumina Hiseq PE150 (Illumina Corporation, San Diego, California, USA) using NEBNext Multiplex Oligos for Illumina (New England BioLabs, Ipswich, Massachusetts, USA). To validate and determine nucleotide sequences of eight gene segments of influenza A virus, each nucleotide sequence read, 150 bp each (read) was trimmed to remove adaptors. Then nucleotide sequences were assembly using de-novo assembly method by CLC genomics workbench software Version 11.0.1. (QIAGEN; Hilden, Germany). The sequence contigs were compared with sequence database by BLAST. After the references influenza viruses were selected from BLAST results, the trimmed sequences were used for read mapping to references. Finally, the whole genome sequences were extracted to FASTA format (.fas) by CLC genomics workbench software. Nucleotide sequences of 8 gene segments of swine influenza virus were submitted to the GenBank database under accession number # MT377918-MT378109 (Table 1).

For phylogenetic analysis, the nucleotide sequences of each gene of S-IAVs from this study were compared with reference S-IAVs. The reference nucleotide sequences of S-IAVs were obtained from Influenza Research Database (https://www.fludb.org). The reference S-IAVs were selected to represent clades/clusters, geographic locations and times of isolation of the viruses. For H1 gene, the reference viruses include the S-IAVs of Classical swine lineage, CS (1A), human seasonal lineage, Hu (1B), Eurasian avian-like swine lineage, EA (1C). The Classical swine lineage (1A) can be further divided into 6 sub-clusters: alpha (1A.1), beta (1A.2), gamma1 (1A.3.3.3), gamma2 (1A.3.2), pdm09 (1A.3.3.2) and North American triple reassortant (TR) lineage lineages^3^. For HA3 gene, the reference viruses include the S-IAVs of North America lineage and Eurasian swine lineage. The North American lineage contains 4 clades of S-IAVs (Clade I, II, III, IV). The Clade IV of North American S-IAV can be further divided into 6 sub-clusters: A-F. For NA1 gene, the reference viruses include the S-IAVs of Classical swine lineage (CS), human seasonal lineage (Human seasonal), North American triple reassortant (TR) lineage and Eurasian avian-like swine lineage (EA). For NA2 gene, the reference viruses include the S-IAVs of North American and Eurasian swine lineages. The North American lineage can be further divided into 2 sub-groups based on year: 1998 from earlier H3N2 introduction (N2-1998–2012) and 2002 from 2000s human seasonal H1 introduction (N2-2012–2016). For internal genes, the reference viruses include the S-IAVs of Classical swine lineage, human seasonal lineage, North American triple reassortant and Eurasian swine lineage.

To construct phylogenetic trees, the nucleotide sequences of each gene of the viruses (both references viruses and viruses form this study) were aligned with Muscle program v3.6^34^ in MEGA v7.0 software^35^. Phylogenetic analysis of HA and NA gene of S-IAV was performed by using a BEAST 2.0 program applying a Bayesian Markov Chain Monte Carlo (BMCMC) algorithm. The best-fit substitution model was implemented by bModelTest (Bayesian model test package for BEAST 2). A strict clock model with coalescent constant population was used as model parameters. The Bayesian MCMC chain lengths were 50,000,000 generations, with sampling every 10,000 generations. The tree iteration was discharged with 10% of the chains as a burn-in pattern by using a tree annotator. The resulting MCC tree was drawn with FigTree software (v1.4.2) (Molecular evolution, phylogenetic and epidemiology, Edinburgh, Scotland, UK). Posterior probability and times of most recent common ancestor (TMRCA) among S-IAVs are provided on branches of trees. Phylogenetic analysis of each internal gene of S-IAV was performed by using MEGA v.7.0 (Tempe, AZ, USA) with neighbor-joining method with Kimura 2-parameter with 1000 bootstrap replicates and Beast program with Bayesian Markov chain Monte Carlo (BMCMC) with 50,000,000 generations and an average standard deviation of split frequencies < 0.05. Substitution rates among sites were set in uniform rate and gabs in the sequences were treated in pairwise deletion. To assign genotype of the S-IAVs, lineages or clusters of each gene of the virus were assigned based on the comparison to reference viruses. After lineages or clusters of gene are assigned, the combination of eight lineages or clusters was assigned as genotype or genetic constellation of S-IAVs. The genotypes of the S-IAVs can help to identify reassortment and genetic diversity of the viruses.

### Serological test for antibodies against swine influenza virus

Hemagglutination inhibitor test (HI test) was used for detecting antibodies against S-IAVs. In this study, HI test was performed to detect antibodies against 3 reference antigens including endemic S-IAV-H1N1 (A/swine/Thailand/CU-CB1/06), pandemic H1N1-2009 (A/swine/Thailand/CU-RA29/2009) and endemic S-IAV-H3N2 (A/swine/Thailand/CU-CB8.4/2007). In detail, the serum sample was incubated at 56 °C for 30 min to remove heat-labile non-specific factor. Then 100 µl of serum sample was treated to remove non-specific inhibitor. For HI test of S-IAV-H3N2 virus, receptor destroying enzyme (RDE) was used to treat serum (50 µl of serum: 150 µl of RDE) and incubated at 37 °C for 24 h and inactivated at 56 °C for 1 h. The RDE-treated serum was mixed with 100 µl of 50% chicken erythrocyte and incubated at room temperature for 1 h. After centrifuging at 2000 rpm for 10 min, the supernatant was free from non-specific inhibitor serum. For HI test of S-IAV-H1N1 and pdmH1N1-2009, 20% of kaolin was used to treat serum (100 µl of serum: 400 µl of 20% kaolin) and incubated at 25 °C for 30 min. For sedimentation of kaolin, the kaolin-treated serum was centrifuged at 2000 rpm for 10 min. The serum was added with 100 µl of 50% chicken erythrocyte and incubated at 25 °C for 1 h. After centrifuging at 2000 rpm for 10 min, the supernatant was free from non-specific inhibitor serum. To perform HI test, reference antigens were prepared to 8 HA unit per 50 µl. First 50 µl of treated serum was mixed with 50 µl of PBS in the first well and serially twofold diluted. 50 µl of reference serum was added in each well and incubated at 25 °C for 45 min. 100 µl of 0.5% chicken erythrocyte was added into each well. After incubating at 25 °C for 1 h, the samples with HI titer of ≥ 1:40 were interpreted as positive against specific S-IAVs subtypes.

### Ethics statement

This study was conducted under the approval of the Institute for Animal Care and Use Protocol of the Chulalongkorn University (CU-VET; IACUC#1831103). All animal study procedures were performed in accordance with CU-VET IACUC guidelines and regulations.

## Supplementary information

Supplementary Information 1.

Supplementary Information 2.

## Data Availability

The nucleotide sequence data that support the findings of this study are openly available in the GenBank database at https://www.ncbi.nlm.nih.gov/genbank/, accession numbers # MT377918-MT378109.
