# Supplementary material for: Persistence of pdm2009-H1N1 internal genes of swine influenza in pigs, Thailand
Source: Sci Rep. 2020 Nov 16;10:19847. doi: 10.1038/s41598-020-76771-2 (PMC7669897; doi:10.1038/s41598-020-76771-2)
Supplement: Supplementary file 1 — Supplementary Information 1. [file 41598_2020_76771_MOESM1_ESM.docx]

**Supplement Table**

**Persistence of pdm2009-H1N1 internal genes of swine influenza in pigs, Thailand**

Chanakarn Nasamran^1,2^, Taveesak Janetanakit^1,2^, Supasama Chiyawong^1,2^,

Supanat Boonyapisitsopa^1,2^, Napawan Bunpapong^1,2^ , Duangduean Prakairungnamthip^1,3^,

Aunyaratana Thontiravong^1,3^, Alongkorn Amonsin^1,2*^

**Author affiliations:**

^1^ Center of Excellence for Emerging and Re-emerging Infectious Diseases in Animals,

Faculty of Veterinary Science, Chulalongkorn University, Bangkok, Thailand

^2^ Department of Veterinary Public Health, Faculty of Veterinary Science, Chulalongkorn University, Bangkok, Thailand

^3^ Department of Microbiology, Faculty of Veterinary Science, Chulalongkorn University, Bangkok, Thailand

*Corresponding author: Professor Dr. Alongkorn Amonsin

Mailing address: Department of Veterinary Public Health, Faculty of Veterinary Science, Chulalongkorn University, Bangkok, Thailand 10330

Phone: +66 2218 9578 Fax: +66 2218 9577

E-mail: Alongkorn.a@chula.ac.th

**Supplement Table 1.** Description of pig sample collection, S-IAV detection, S-IAV isolation and serological results in this study

| Month | Species | # Nasal swabs | PCR  IAV positive  (% positive) | Virus isolation  IAV positive  (% positive) | # Serum  samples | HI titer* | | |
| --- | --- | --- | --- | --- | --- | --- | --- | --- |
|  |  |  |  |  |  | S-H1N1 | S-H3N2 | pdmH1N1 |
| Jan-17 | Swine | 30 | 8/30 (26.67%) | 7/30 (23.33%) | 30 | 2/30 (6.67%) | 7/30 (23.33%) | 0/30 (0%) |
| Apr-17 | Swine | 30 | 5/30 (16.67%) | 4/30 (13.33%) | 30 | 3/30 (10%) | 22/30 (73.33%) | 4/30 (13.33%) |
| Jun-17 | Swine | 30 | 10/30 (33.33%) | 11/30 (36.67%) | 30 | 2/30 (6.67%) | 19/30 (63.33%) | 1/30 (3.33%) |
| Dec-17 | Swine | 30 | 8/30 (26.67%) | 3/30 (10%) | 30 | 5/30 (16.67%) | 13/30 (43.33%) | 15/30 (50%) |
| Feb-18 | Swine | 31 | 1/31 (3.23%) | 0/31 (0%) | 31 | 4/31 (12.9%) | 14/31 (45.16%) | 6/31 (19.35%) |
| Mar-18 | Swine | 34 | 0/34 (0%) | 0/34 (0%) | 34 | 5/34 (14.71%) | 3/34 (8.82%) | 13/34 (38.24%) |
| Apr-18 | Swine | 32 | 13/32 (40.63%) | 14/32 (43.75%) | 32 | 7/32 (21.88%) | 8/32 (25%) | 4/32 (12.5%) |
| May-18 | Swine | 31 | 10/31 (32.26%) | 2/31 (6.45%) | 31 | 3/31 (9.68%) | 9/31 (29.03%) | 12/31 (38.71%) |
| Jun-18 | Swine | 30 | 3/30 (10%) | 2/30 (6.67%) | 30 | 5/30 (16.67%) | 3/30 (10%) | 4/30 (13.33%) |
| Jul-18 | Swine | 30 | 4/30 (13.33%) | 1/30 (3.33%) | 30 | 2/30 (6.67%) | 13/30 (43.33%) | 19/30 (63.33%) |
| Aug-18 | Swine | 31 | 6/31 (19.35%) | 1/31 (3.23%) | 31 | 9/31 (29.03%) | 10/31 (32.26%) | 15/31 (48.39%) |
| Sep-18 | Swine | 33 | 3/33 (9.09%) | 4/33 (12.12%) | 33 | 6/33 (18.18%) | 17/33 (51.52%) | 4/33 (12.12%) |
| Oct-18 | Swine | 32 | 5/32 (15.63%) | 5/32 (15.63%) | 32 | 6/32 (18.75%) | 1/32 (3.13%) | 4/32 (12.5%) |
| Nov-18 | Swine | 32 | 6/32 (18.75%) | 3/32 (9.38%) | 32 | 10/32 (31.25%) | 1/32 (3.13%) | 1/32 (3.13%) |
|  |  | 436 | 82/436 (18.81%) | 57/436 (13.07%) | 436 | 69/436 (15.83%) | 140/436 (32.11%) | 102/436 (23.39%) |

* HI antibody titer against; S-H1N1; endemic S-IAV-H1N1 (A/swine/Thailand/CU-CB1/06), pdmH1N1; pandemic H1N1-2009 (A/swine/Thailand/CU-RA29/2009) and S-H3N2; endemic S-IAV-H3N2 (A/swine/Thailand/CU-CB8.4/2007)

**Supplement Table 2**. Gene constellation of Thai H1N1 swine influenza viruses in this study

| Viruses | Genotype | Date | Gene segments | | | | | | | |
| --- | --- | --- | --- | --- | --- | --- | --- | --- | --- | --- |
|  |  |  | PB2 | PB1 | PA | HA | NP | NA | M | NS |
| SIV-H1N1 |  |  |  |  |  |  |  |  |  |  |
| THA/CUPL65/2010 | pdm | 2010 | pdm | pdm | pdm | pdm | pdm | pdm | pdm | pdm |
| THA/NIAH587/2005 | H1N1(7+1) | 2005 | EA | EA | EA | CS | EA | EA | EA | EA |
| THA/CUS3340N/2012 | H1N1(6+2) | 2012 | EA | EA | EA | CS | EA | EA | EA | CS |
| THA/CUSA43/2010 | rH1N1(7+1) | 2010 | pdm | pdm | pdm | pdm | pdm | EA | pdm | pdm |
| THA/CUS3629N/2012 | rH1N1(pdm+2) | 2013 | pdm | pdm | pdm | CS | pdm | EA | pdm | pdm |
| THA/CU3732/2017 | rH1N1(pdm+2) | Jan-17 | pdm | pdm | pdm | CS | pdm | EA | pdm | pdm |
| THA/CU3743/2017 | rH1N1(pdm+2) | Jan-17 | pdm | pdm | pdm | CS | pdm | EA | pdm | pdm |
| THA/CU3759/2017 | rH1N1(pdm+2) | Apr-17 | pdm | pdm | pdm | CS | pdm | EA | pdm | pdm |
| THA/CU3764/2017 | rH1N1(pdm+2) | Apr-17 | pdm | pdm | pdm | CS | pdm | EA | pdm | pdm |
| THA/CU3770/2017 | rH1N1(pdm+2) | Apr-17 | pdm | pdm | pdm | CS | pdm | EA | pdm | pdm |
| THA/CU3793/2017 | rH1N1(pdm+2) | Jul-17 | pdm | pdm | pdm | CS | pdm | EA | pdm | pdm |
| THA/CU3802/2017 | rH1N1(pdm+1) | Jul-17 | pdm | pdm | pdm | CS | pdm | pdm | pdm | pdm |
| THA/CU3796/2017 | rH1N1(pdm+1) | Jul-17 | pdm | pdm | pdm | CS | pdm | pdm | pdm | pdm |
| THA/CU3803/2017 | rH1N1(pdm+1) | Jul-17 | pdm | pdm | pdm | CS | pdm | pdm | pdm | pdm |
| THA/CU21299/2018 | rH1N1(pdm+1) | Apr-18 | pdm | pdm | pdm | CS | pdm | pdm | pdm | pdm |
| THA/CU21304/2018 | rH1N1(pdm+1) | Apr-18 | pdm | pdm | pdm | CS | pdm | pdm | pdm | pdm |
| THA/CU21626/2018 | rH1N1(pdm+1) | Jun-18 | pdm | pdm | pdm | CS | pdm | pdm | pdm | pdm |
| THA/CU21630/2018 | rH1N1(pdm+1) | Jun-18 | pdm | pdm | pdm | CS | pdm | pdm | pdm | pdm |
| THA/CU21970/2018 | rH1N1(pdm+1) | Jul-18 | pdm | pdm | pdm | CS | pdm | pdm | pdm | pdm |
| THA/CU22117/2018 | rH1N1(pdm+1) | Aug-18 | pdm | pdm | pdm | CS | pdm | pdm | pdm | pdm |
| THA/CU22300/2018 | rH1N1(pdm+1) | Sep-18 | pdm | pdm | pdm | CS | pdm | pdm | pdm | pdm |
| THA/CU22351/2018 | rH1N1(pdm+1) | Oct-18 | pdm | pdm | pdm | CS | pdm | pdm | pdm | pdm |
| THA/CU22630/2018 | rH1N1(pdm+1) | Nov-18 | pdm | pdm | pdm | CS | pdm | pdm | pdm | pdm |

| Viruses | Genotype | Year | Gene segment | | | | | | | |
| --- | --- | --- | --- | --- | --- | --- | --- | --- | --- | --- |
|  |  |  | PB2 | PB1 | PA | HA | NP | NA | M | NS |
| SIV-H3N2 |  |  |  |  |  |  |  |  |  |  |
| THA/KU5.1/2004 | eH3N2 | 2004 | EA | EA | EA | Hu | CS | Hu | EA | CS |
| THA/CUS3673N/2012 | rH3N2(pdm+2) | 2013 | pdm | pdm | pdm | Hu | pdm | Hu | pdm | pdm |
| THA/CU3794/2017 | rH3N2(pdm+2) | Jul-17 | pdm | pdm | pdm | Hu | pdm | Hu | pdm | pdm |
| THA/CU3816/2017 | rH3N2(pdm+2) | Jul-17 | pdm | pdm | pdm | Hu | pdm | Hu | pdm | pdm |
| THA/CU3790/2017 | rH3N2(pdm+2) | Jul-17 | pdm | pdm | pdm | Hu | pdm | Hu | pdm | pdm |
| THA/CU20226/2017 | rH3N2(pdm+2) | Dec-17 | pdm | pdm | pdm | Hu | pdm | Hu | pdm | pdm |
| THA/CU20218/2017 | rH3N2(pdm+2) | Dec-17 | pdm | pdm | pdm | Hu | pdm | Hu | pdm | pdm |
| THA/CU22337/2018 | rH3N2(pdm+2) | Oct-18 | pdm | pdm | pdm | Hu | pdm | Hu | pdm | pdm |
| pdm: pdm09 lineage, CS: Classical swine lineage, EA: Eurasian avian-like lineage, Hu: Human-like swine | | | | | | | | | | |
