# Supplementary material for: Persistence of pdm2009-H1N1 internal genes of swine influenza in pigs, Thailand
Source: Sci Rep. 2020 Nov 16;10:19847. doi: 10.1038/s41598-020-76771-2 (PMC7669897; doi:10.1038/s41598-020-76771-2)

**Supplement Figures**

**Persistence of pdm2009-H1N1 internal genes of swine influenza in pigs, Thailand**

Chanakarn Nasamran^1,2^, Taveesak Janetanakit^1,2^, Supasama Chiyawong^1,2^,

Supanat Boonyapisitsopa^1,2^, Napawan Bunpapong^1,2^ , Duangduean Prakairungnamthip^1,3^,

Aunyaratana Thontiravong^1,3^, Alongkorn Amonsin^1,2*^

**Author affiliations:**

^1^ Center of Excellence for Emerging and Re-emerging Infectious Diseases in Animals,

Faculty of Veterinary Science, Chulalongkorn University, Bangkok, Thailand

^2^ Department of Veterinary Public Health, Faculty of Veterinary Science, Chulalongkorn University, Bangkok, Thailand

^3^ Department of Microbiology, Faculty of Veterinary Science, Chulalongkorn University, Bangkok, Thailand

*Corresponding author: Professor Dr. Alongkorn Amonsin

Mailing address: Department of Veterinary Public Health, Faculty of Veterinary Science, Chulalongkorn University, Bangkok, Thailand 10330

Phone: +66 2218 9578 Fax: +66 2218 9577

E-mail: Alongkorn.a@chula.ac.th

**Supplement Figures**

**Supplement Figure 1.** Phylogenetic tree of PB2 gene of S-IAV-H1N1 and S-IAV-H3N2. Circles indicate S-IAV-H1N1 isolated in this study. The phylogenetic tree was generated by neighbor- joining method with Kimura 2-parameter with 1,000 bootstrap replicates and Beast program with Bayesian Markov chain Monte Carlo (BMCMC) with 50,000,000 generations and an average standard deviation of split frequencies <0.05. Bootstrap value (>70% %) and posterior probability (>0.70) values are shown on branches to support tree topology.

**Supplement Figure 2.** Phylogenetic tree of PB1 gene of S-IAV-H1N1 and S-IAV-H3N2. Circles indicate S-IAV-H1N1 isolated in this study. The phylogenetic tree was generated by neighbor- joining method with Kimura 2-parameter with 1,000 bootstrap replicates and Beast program with Bayesian Markov chain Monte Carlo (BMCMC) with 50,000,000 generations and an average standard deviation of split frequencies <0.05. Bootstrap value (>70% %) and posterior probability (>0.70) values are shown on branches to support tree topology.

**Supplement Figure 3.** Phylogenetic tree of PA gene of S-IAV-H1N1 and S-IAV-H3N2. Circles indicate S-IAV-H1N1 isolated in this study. The phylogenetic tree was generated by neighbor- joining method with Kimura 2-parameter with 1,000 bootstrap replicates and Beast program with Bayesian Markov chain Monte Carlo (BMCMC) with 50,000,000 generations and an average standard deviation of split frequencies <0.05. Bootstrap value (>70% %) and posterior probability (>0.70) values are shown on branches to support tree topology.

**Supplement Figure 4.** Phylogenetic tree of NP gene of S-IAV-H1N1 and S-IAV-H3N2. Circles indicate S-IAV-H1N1 isolated in this study. The phylogenetic tree was generated by neighbor- joining method with Kimura 2-parameter with 1,000 bootstrap replicates and Beast program with Bayesian Markov chain Monte Carlo (BMCMC) with 50,000,000 generations and an average standard deviation of split frequencies <0.05. Bootstrap value (>70% %) and posterior probability (>0.70) values are shown on branches to support tree topology.

**Supplement Figure 5.** Phylogenetic tree of M gene of S-IAV-H1N1 and S-IAV-H3N2. Circles indicate S-IAV-H1N1 isolated in this study. The phylogenetic tree was generated by neighbor- joining method with Kimura 2-parameter with 1,000 bootstrap replicates and Beast program with Bayesian Markov chain Monte Carlo (BMCMC) with 50,000,000 generations and an average standard deviation of split frequencies <0.05. Bootstrap value (>70% %) and posterior probability (>0.70) values are shown on branches to support tree topology.

**Supplement Figure 6.** Phylogenetic tree of NS gene of S-IAV-H1N1 and S-IAV-H3N2. Circles indicate S-IAV-H1N1 isolated in this study. The phylogenetic tree was generated by neighbor- joining method with Kimura 2-parameter with 1,000 bootstrap replicates and Beast program with Bayesian Markov chain Monte Carlo (BMCMC) with 50,000,000 generations and an average standard deviation of split frequencies <0.05. Bootstrap value (>70% %) and posterior probability (>0.70) values are shown on branches to support tree topology.

**Supplement Figure 1.**


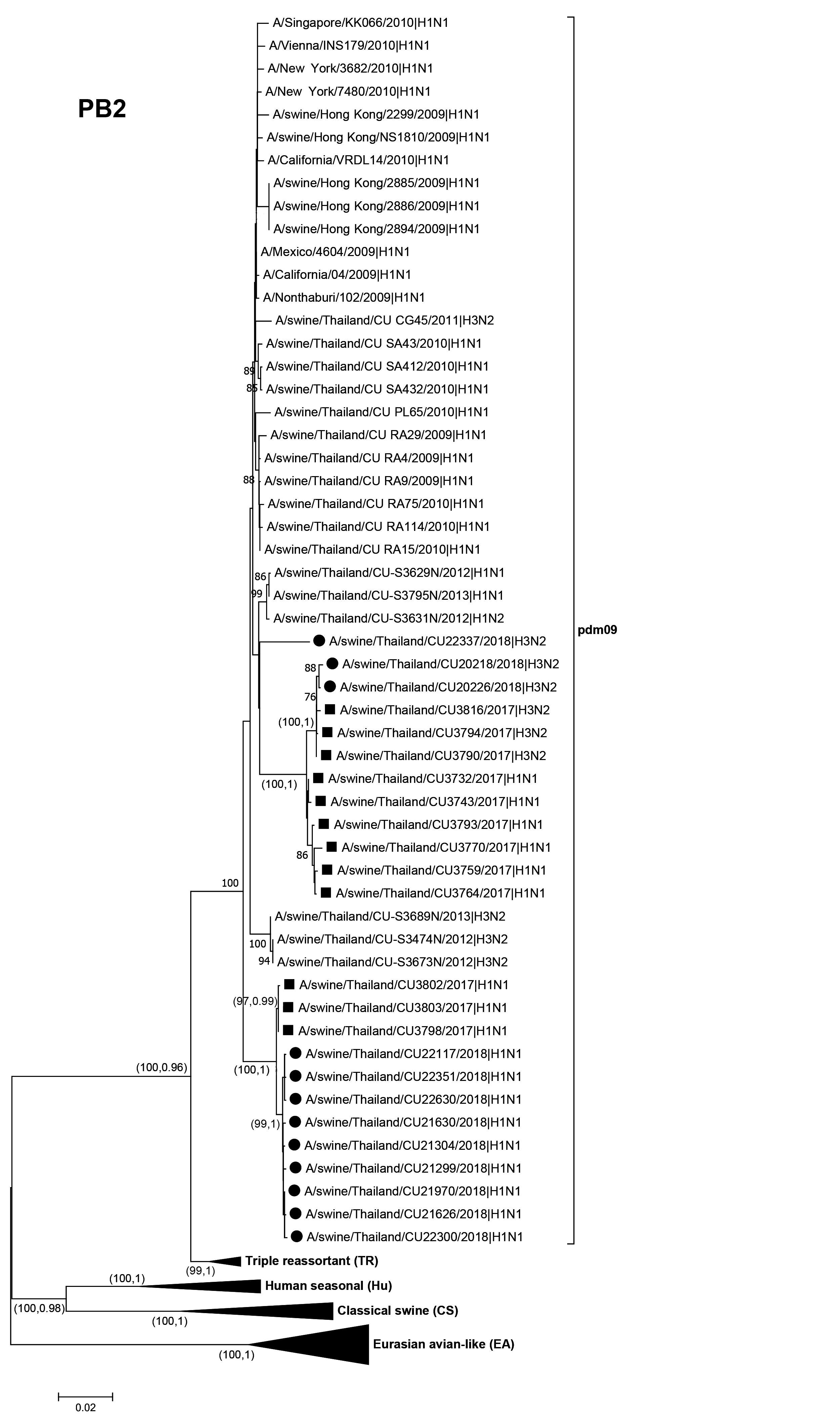


**Supplement Figure 2.**


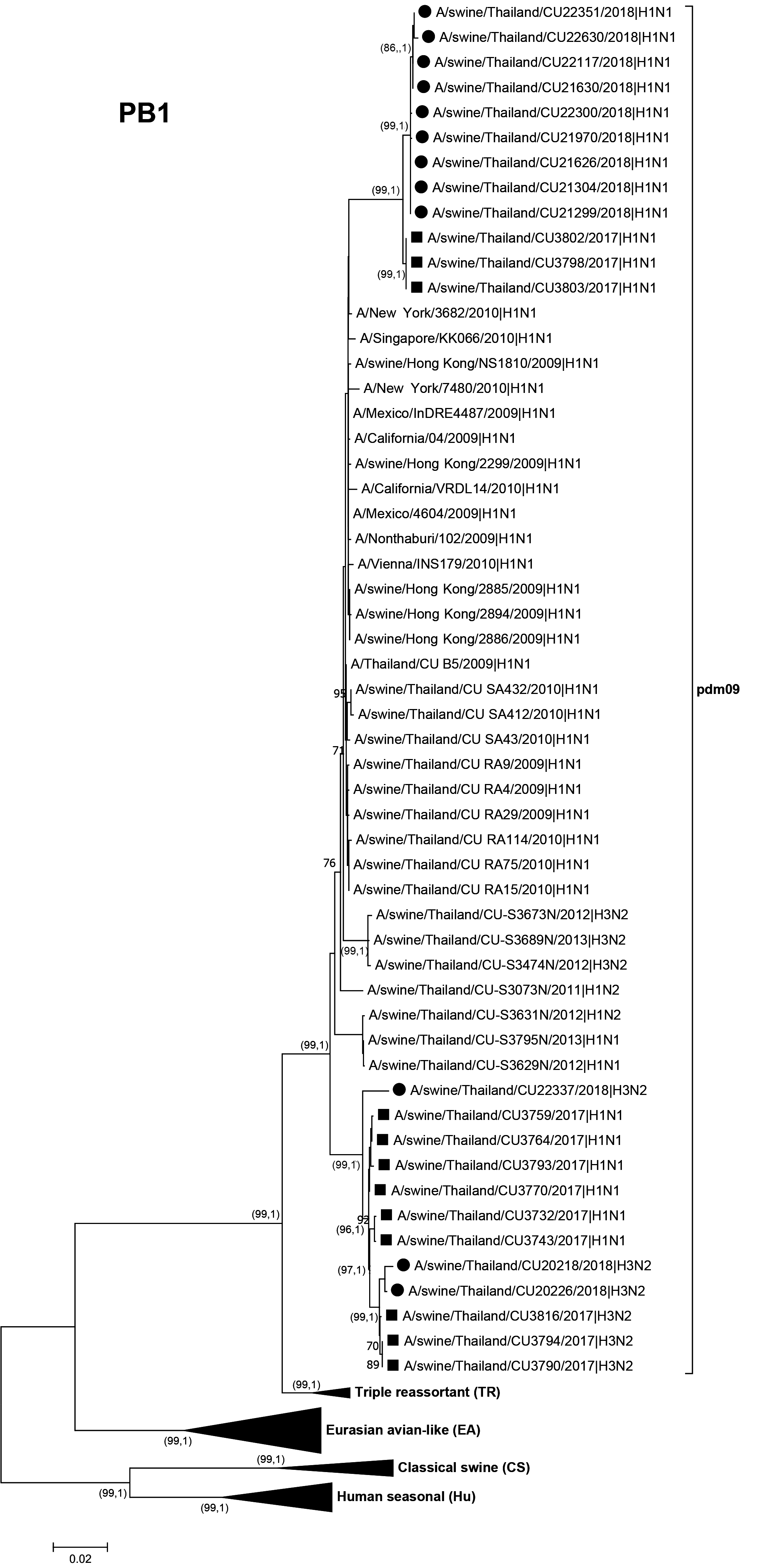


**Supplement Figure 3.**


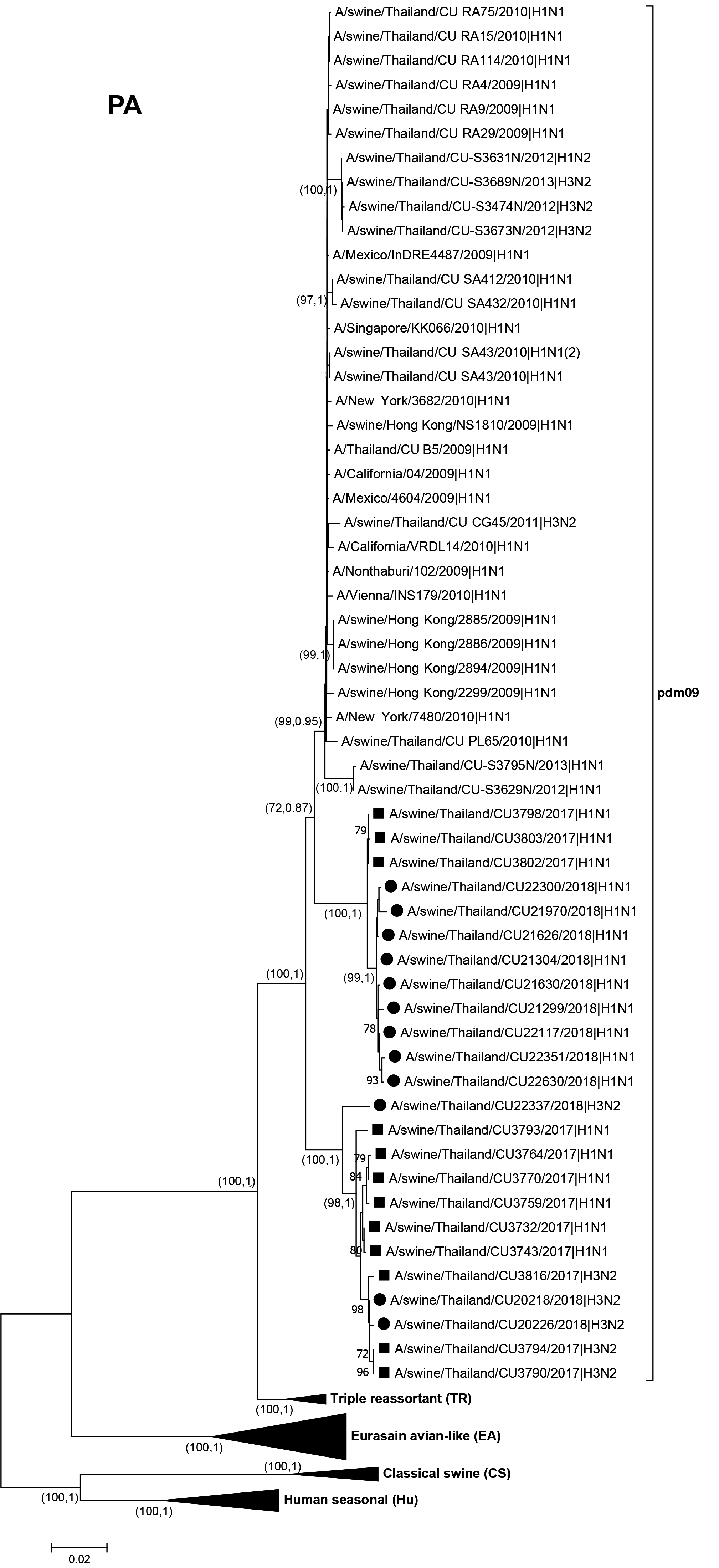


**Supplement Figure 4.**


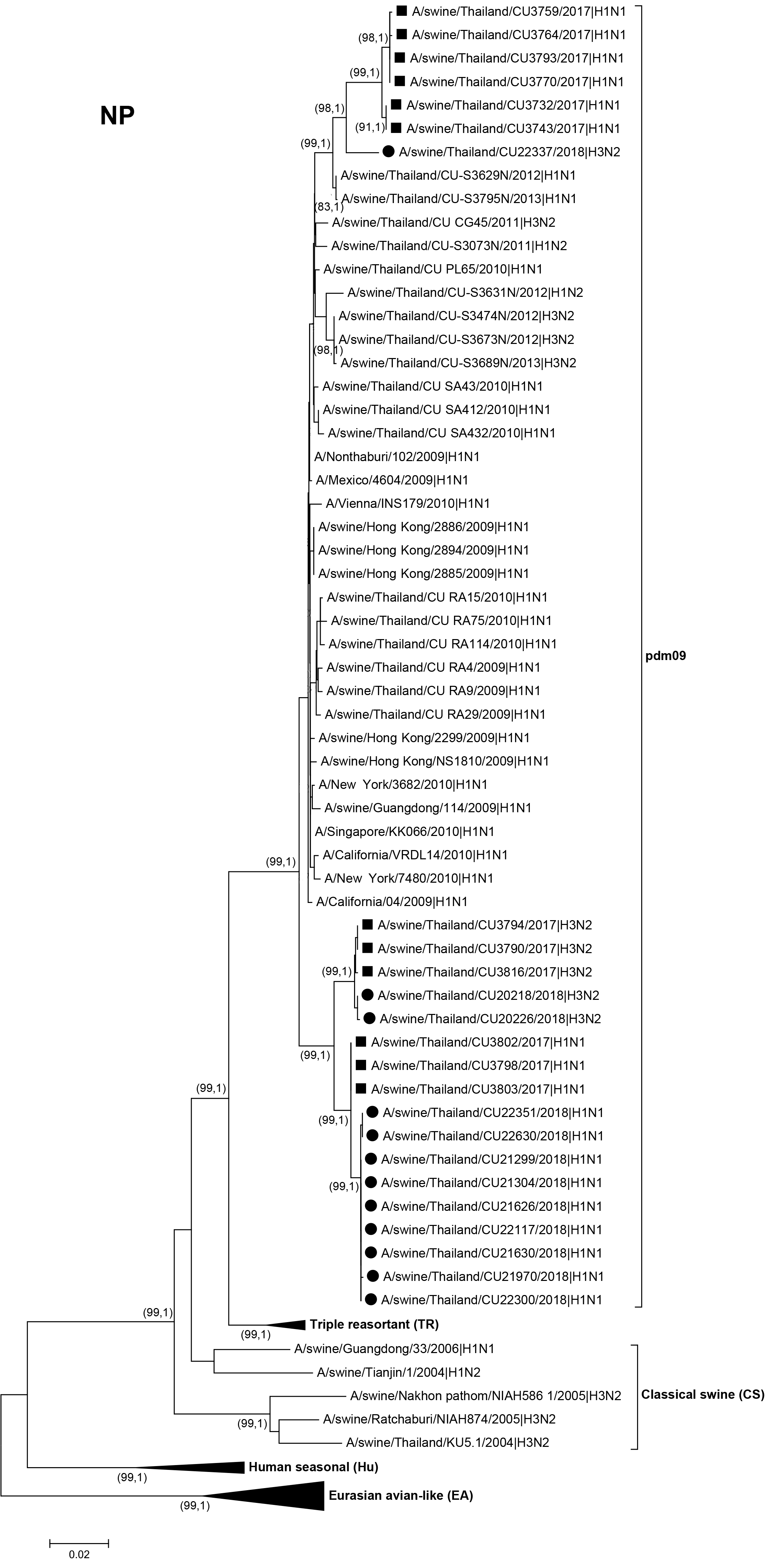


**Supplement Figure 5.**


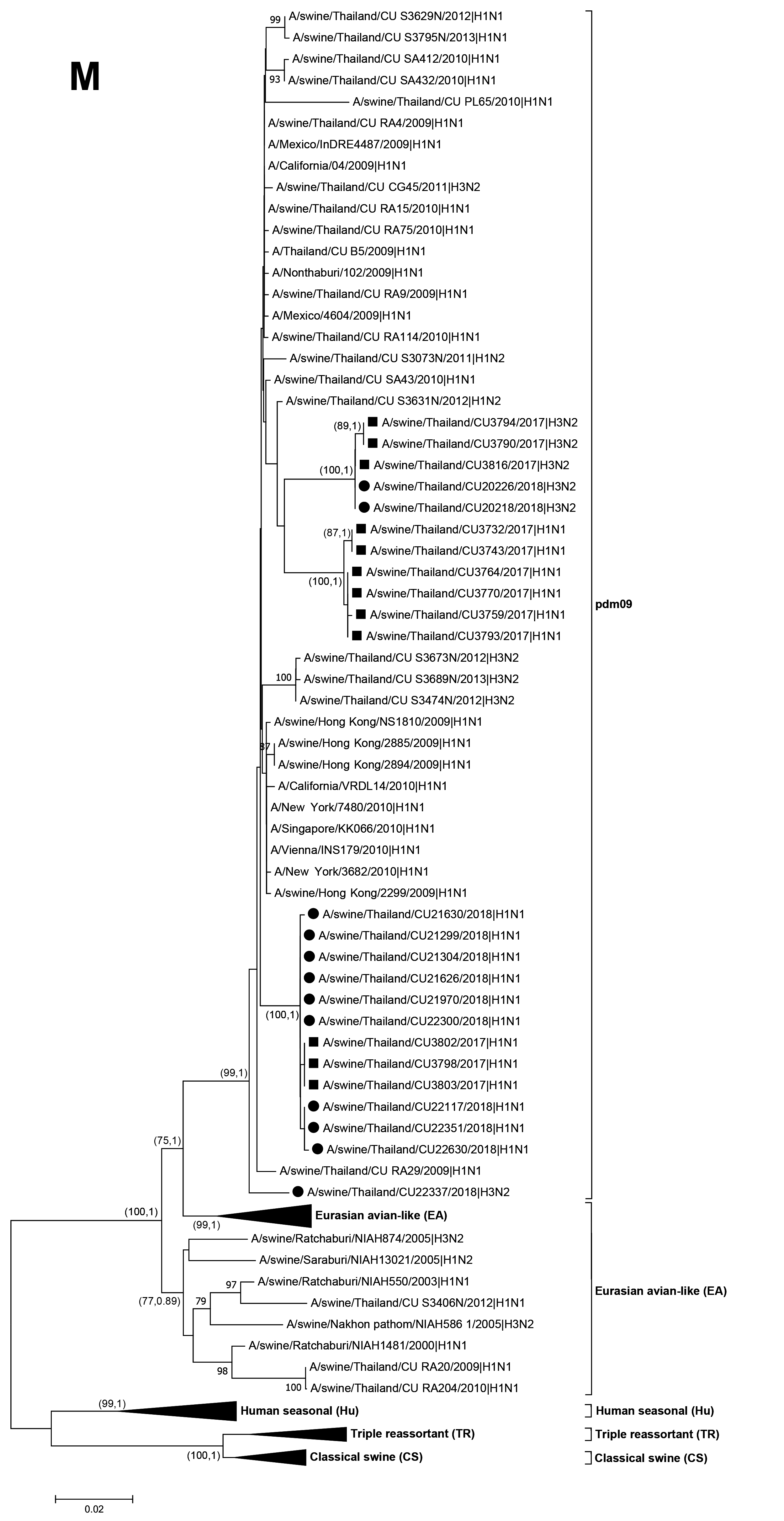


**Supplement Figure 6.**


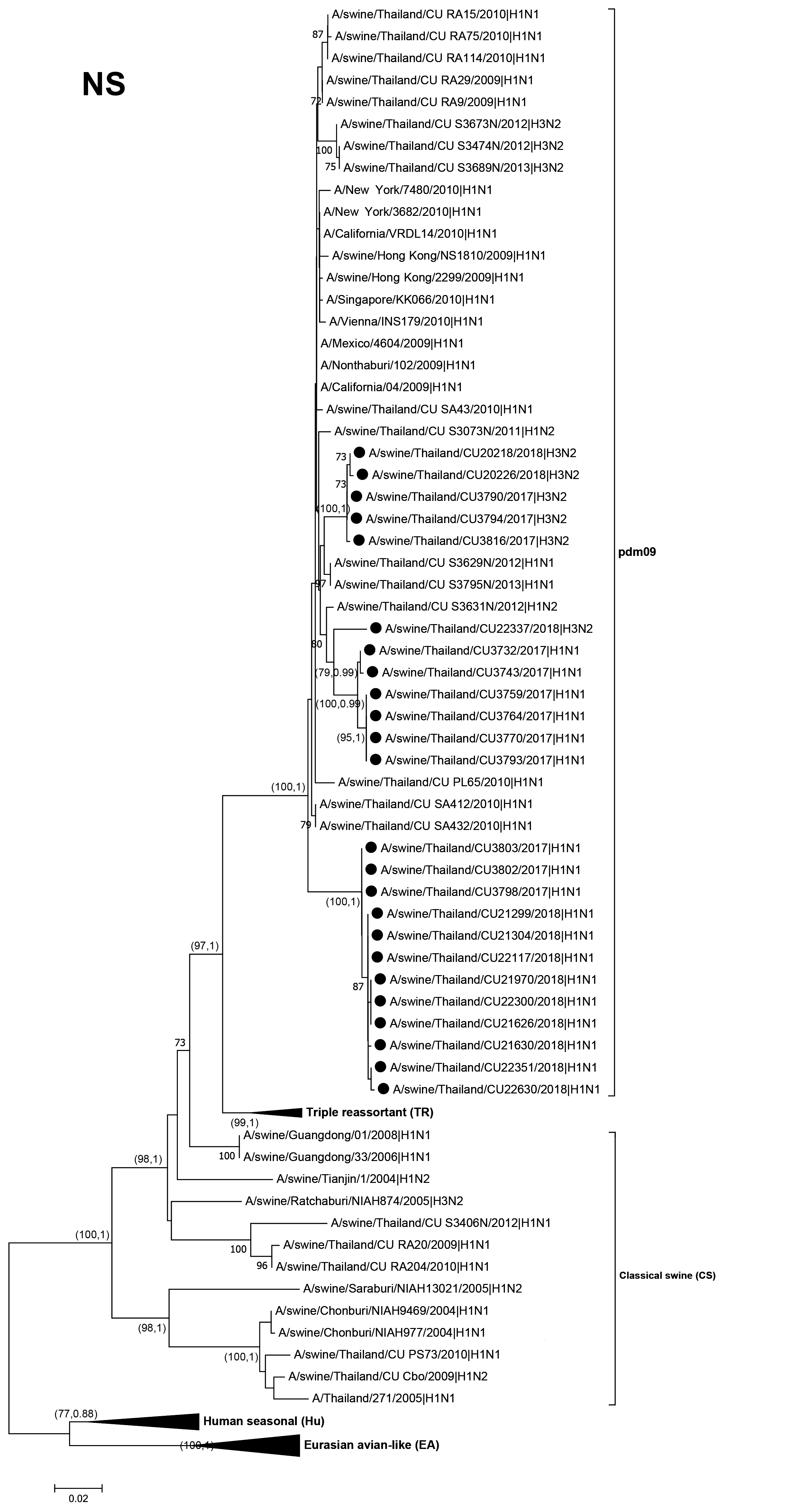

Supplement: Supplementary file 2 — Supplementary Information 2. [file 41598_2020_76771_MOESM2_ESM.docx]
